# Supplementary material for: Ignoring stratigraphic age uncertainty leads to erroneous estimates of species divergence times under the fossilized birth–death process
Source: Proc Biol Sci. 2019 May 8;286(1902):20190685. doi: 10.1098/rspb.2019.0685 (PMC6532507; doi:10.1098/rspb.2019.0685)
Supplement: Supplementary text and figures [file rspb20190685supp1.pdf]

Ignoring stratigraphic age uncertainty leads to erroneous estimates of species divergence times under the fossilized birth-death process
Supplementary Materials

1 Effects of subsampling

To test the effects of subsampling the fossil samples, the Cetacean dataset was also analysed with a subsampling of 5% of the available fossils, i.e 224 fossils.

Figure 1 shows the MCC trees obtained with 5% subsampling when using different age handling methods. These trees follow the same pattern that was obtained in the main analysis, with median ages leading to much older divergence time estimates than interval ages, in particular for the older nodes. However, both the estimated divergence times and the difference in estimates are lower: the MRCA of extant cetaceans is estimated to be 48.5 Ma using median ages, 47 Ma using random ages and 39 Ma using interval ages.

The parameter estimates obtained using 5% subsampling are shown in Figure 2. The diversification rate estimates are slightly higher than the estimates obtained with 10% subsampling, which is consistent with the estimated shorter topology. Since fossil sampling was halved, we expect the sampling proportion to be halved as well: the 5% estimates are actually a bit higher than half the 10% estimates, again consistent with the shorter estimated tree topology. The turnover estimates are very similar between the two analyses.

In conclusion, this analysis shows that different methods of handling fossil age uncertainty lead to differences even when a lower number of fossils are included in the dataset. Although the FBD parameters are only slightly affected by the choice of subsampling, the divergence time estimates show strong differences. Thus the choice of the fossils included in the dataset is also an important consideration when estimating divergence times, although this is outwith the scope of our study.

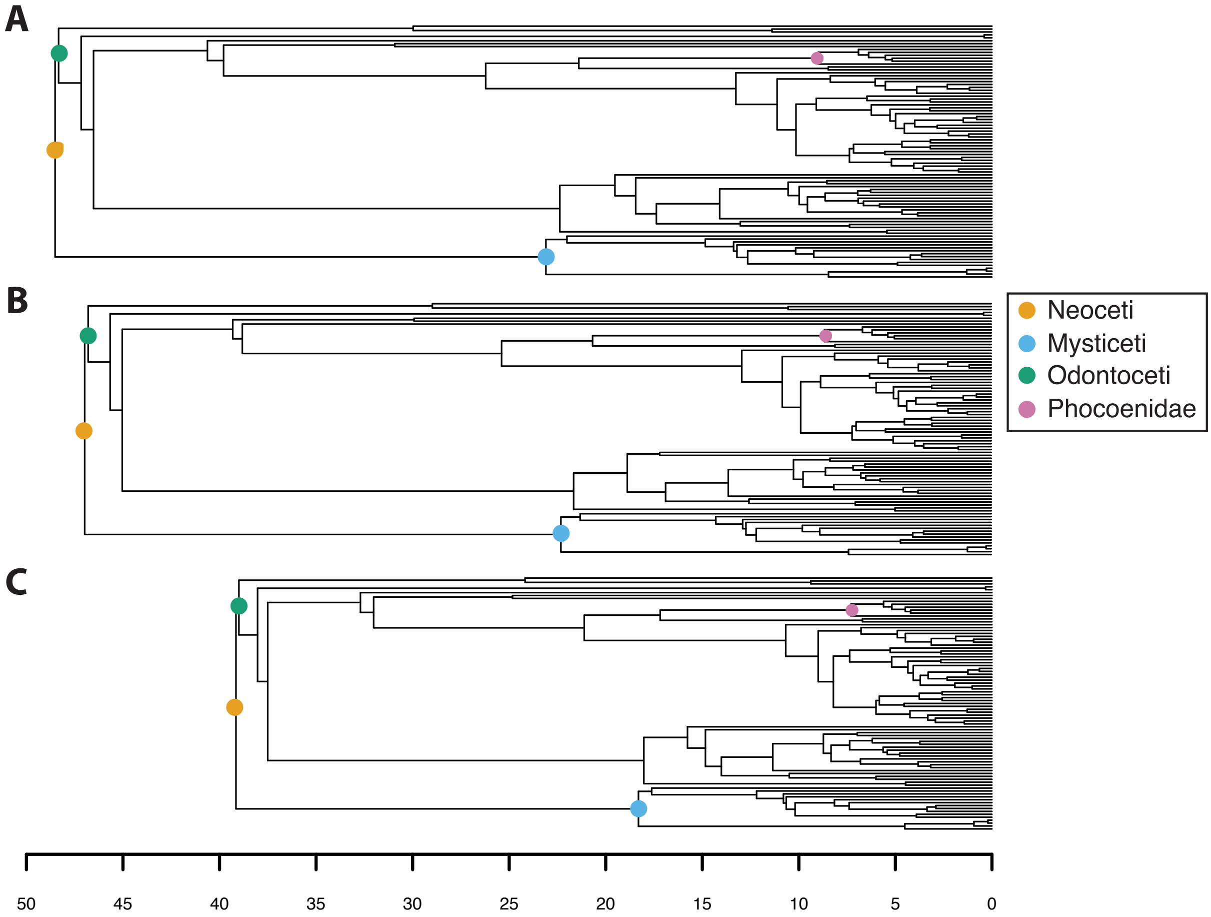

Figure 1: MCC trees inferred for the Cetacea dataset (with 5% subsampling) using the FBD process with fossil ages fixed to median ages (A), random ages (B) or sampled within the known interval of uncertainty (C). The major clades are highlighted.

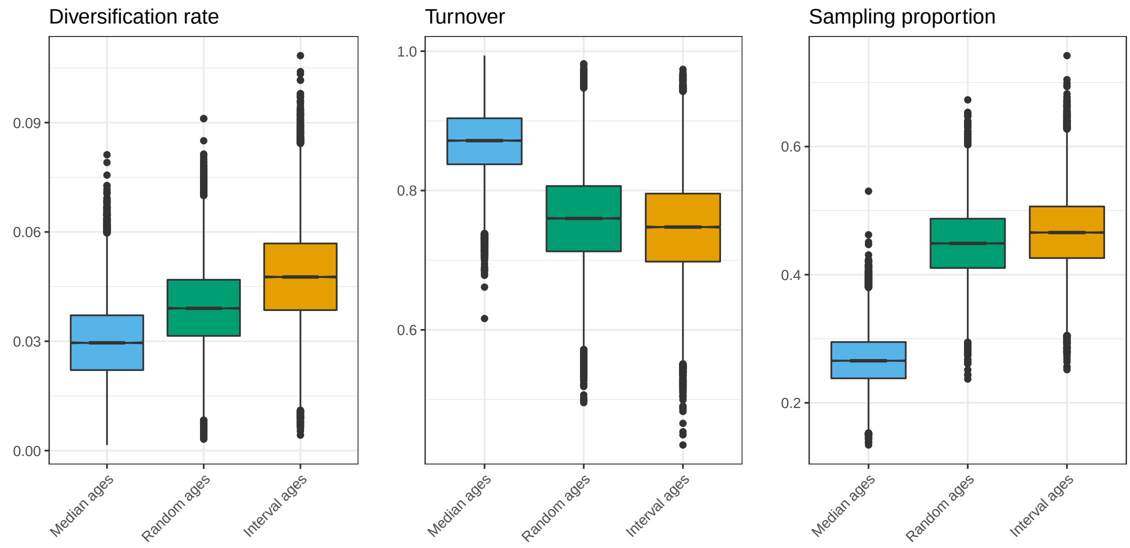

Figure 2: Estimates of the diversification rate, turnover and sampling proportion obtained for the Cetacea dataset (with 5% subsampling) using the FBD process with fossil ages fixed to median ages, random ages or sampled within the known interval of uncertainty.
